# Clinical and radiological evaluation of cage subsidence following oblique lumbar interbody fusion combined with anterolateral fixation

**DOI:** 10.1186/s12891-022-05165-4

**Published:** 2022-03-05

**Authors:** Long Zhao, Tianhang Xie, Xiandi Wang, Zhiqiang Yang, Xingxiao Pu, Yufei Lu, Jiancheng Zeng

**Affiliations:** Department of Orthopedics and Orthopedic Research Institute, West China Hospital, Sichuan University, 37# Wuhou Guoxue road, Chengdu, P.R. China 610041

**Keywords:** Oblique lumbar interbody fusion, Cage subsidence, Clinical effects, Radiological characteristics, Risk factors

## Abstract

**Background:**

Cage subsidence (CS) was previously reported as one of the most common complications following oblique lumbar interbody fusion (OLIF). We aimed to assess the impacts of CS on surgical results following OLIF combined with anterolateral fixation, and determine its radiological characteristics as well as related risk factors.

**Methods:**

Two hundred and forty-two patients who underwent OLIF at L4-5 and with a minimum 12 months follow-up were reviewed. Patients were divided into three groups according to the extent of disk height (DH) decrease during follow-up: no CS (DH decrease ≤ 2 mm), mild CS (2 mm < DH decrease ≤ 4 mm) and severe CS (DH decrease > 4 mm). The clinical and radiological results were compared between groups to evaluate radiological features, clinical effects and risk factors of CS.

**Results:**

CS was identified in 79 (32.6%) patients, including 48 (19.8%) with mild CS and 31 (11.8%) with severe CS. CS was mainly identified within 1 month postoperatively, it did not progress after 3 months postoperatively, and more noted in the caudal endplate (44, 55.7%). In terms of clinical results, patients in the mild CS group were significantly worse than those in the no CS group, and patients in the severe CS group were significantly worse than those in the mild CS group. There was no significant difference in fusion rate between no CS (92.6%, 151/163) and mild CS (83.3%, 40/48) groups. However, significant lower fusion rate was observed in severe CS group (64.5%, 20/31) compared to no CS group. CS related risk factors included osteoporosis (OR = 5.976), DH overdistraction (OR = 1.175), flat disk space (OR = 3.309) and endplate injury (OR = 6.135).

**Conclusion:**

CS following OLIF was an early postoperative complication. Higher magnitudes of CS were associated with worse clinical improvements and lower intervertebral fusion. Osteoporosis and endplate injury were significant risk factors for CS. Additionally, flat disk space and DH over-distraction were also correlated with an increased probability of CS.

## Introduction

Oblique lumbar interbody fusion (OLIF) is an effective treatment for patients with degenerative lumbar disc disease. Indirect decompression of neural elements can be achieved by distracting the reduced intervertebral space with an enlarged interbody cage, thus alleviating neurogenic intermittent claudication [1, 2].

Maintaining the restored intervertebral space is one of the fundamental requirements following OLIF [3]. However, as a complication resulting in loss of the intervertebral space. cage subsidence (CS) has been reported as common event following OLIF, with an incidence rate between 10.0%-40.0% [4, 5].

Currently, variability exists in the impact of CS on surgical outcomes after traditional lumbar interbody fusion (LIF) surgery, with reports of non-adverse events in some studies [6, 7], while other studies report adverse events that cause pain and even failure of surgery [8, 9]. To our knowledge, no early studies have thoroughly addressed whether CS affects surgical outcomes following OLIF. We launched this study to determine the impact of CS on clinical and radiological results following OLIF combined with anterolateral fixation, and to further study its occurrence characteristics and related risk factors, so as to provide recommendations for preventing CS.

## Methods

This was a retrospective study that was approved by the institutional review board in our hospital. Patients who underwent OLIF combined with anterolateral fixation between October 2017 and December 2019 at our institution were retrospectively reviewed, and waived the requirements for informed patients consent because of its retrospective nature. The inclusion criteria were patients who were diagnosed with mild spinal stenosis (Schizas grade A or B [10]) and degenerative instability at L4-5. We excluded patients who underwent surgery at multiple levels, who were diagnosed with severe stenosis (Schizas grade C or D) or stenosis caused by extruded herniated disc, calcified disc or bony spur formation, who were diagnosed with isthmic spondylolisthesis or severe degenerative spondylolisthesis (Meyerding grade II-IV). Patients who had follow-up of less than 12 months were also excluded. Measurements were independently performed by two authors and the mean value was used.

### Surgical procedure

After general anaesthesia intubation, a right lateral decubitus position was taken. A 6-cm skin incision was made in the left lateral abdominal region parallel to the iliac crest. The external oblique, internal oblique, and transverse abdominal muscles were dissected along their fibres, and then a 22-mm-diameter tubular retractor was attached after reaching the index disc. Subsequently, discectomy and endplate preparing were performed. The cartilaginous endplate was removed using reamer and curette until minor bleeding was identified. Then, an appropriate polyetheretherketone cage (Clydesdale Spinal System, Medtronic Sofamor Danek USA, Inc.) (height: 8–14 mm, length: 45–55 mm, width:18 mm, lordotic angle: 6°), whose size was determined by sequential trail implant testing, was filled with the recombined human Bone Morphogenetic Protein-2 (CPC rhBMP-2, Rebone, Shanghai, P.R.C.) and inserted. Next, two screws (length: 40 mm-55 mm, diameter: 6.5 mm) (Medtronic Sofamor Danek USA, Inc) were fixed at the lateral side of the vertebrae close to endplate and locked using a single rod (length: 38 mm-42 mm, diameter: 5.5 mm). Finally, abdominal muscle and incised skin were closed.

### Radiological and clinical evaluation

**Fig. 1:**
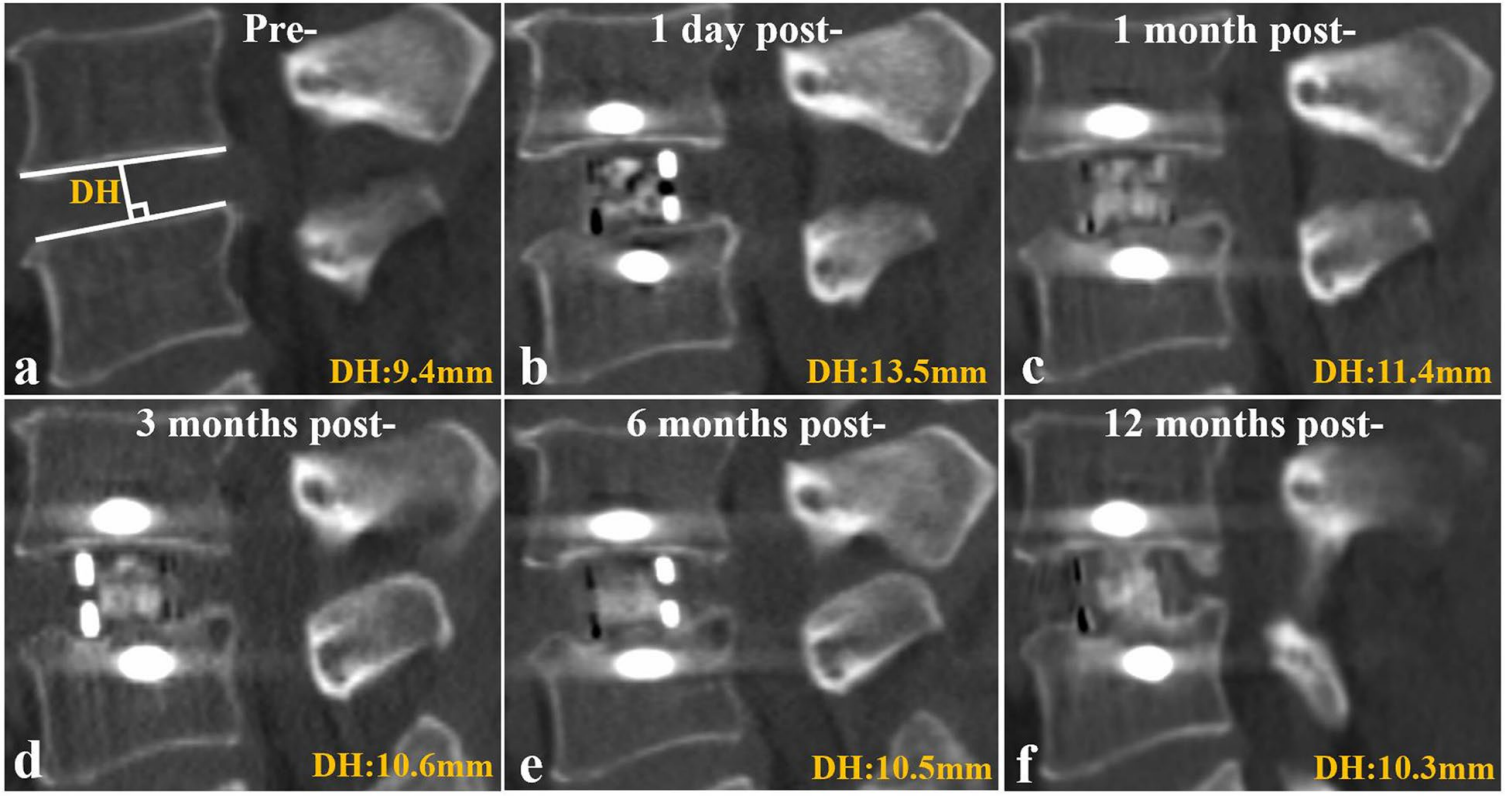
Cage subsidence (CS) course of a 63 years old man. Disk height (DH) was measured as the vertical distance between the midpoint of the cranial endplate and the caudal endplate on the CT midsagittal plane (**a**). CS was identified at 1 month post- (**c**), and obviously progressed at 3 months post- (**d**), while did not significantly change at 6 (**e**) and 12 (**f**) months post-. Pre-, preoperative; post-, postoperative

**Fig. 2:**
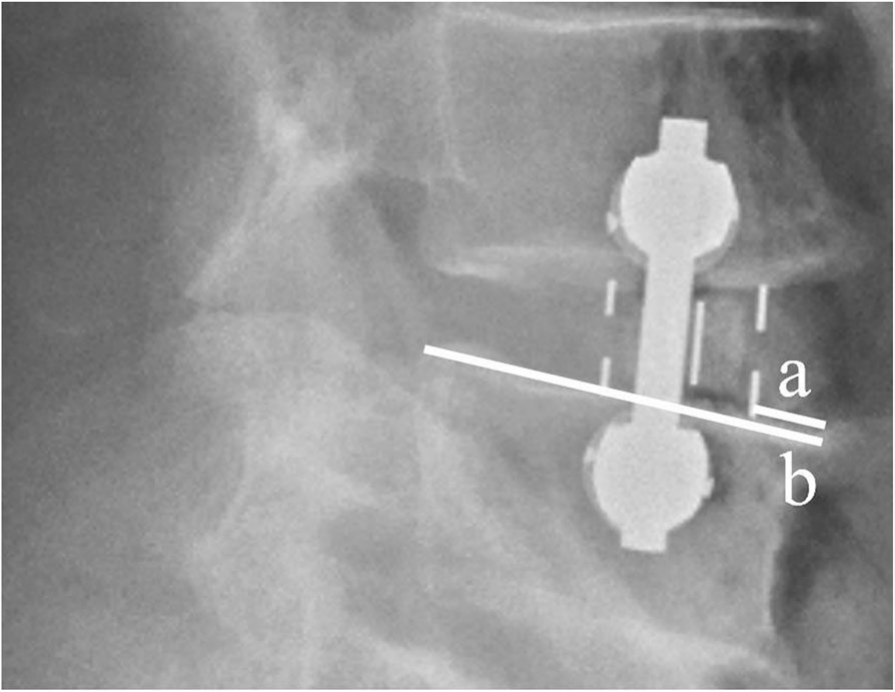
The measurement of cage position. The cage position is calculated as a/b*100%. **a** the distance between the anterior metal marker and the leading edge of the caudal endplate. **b** the length of caudal endplate

**Fig. 3:**
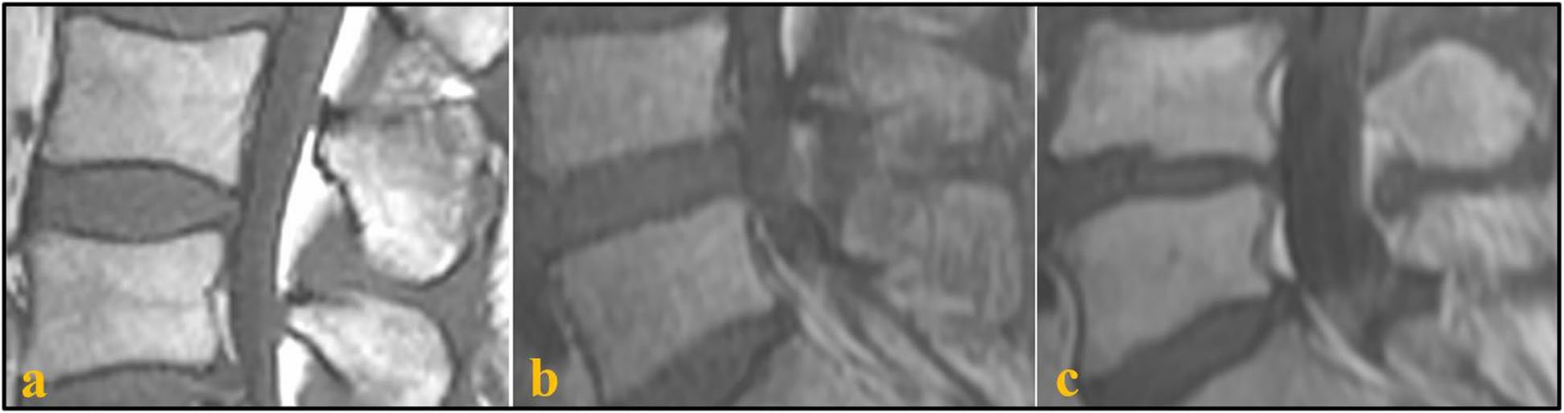
The classification of the disk space morphology on MRI. **a** concave. **b** flat.** c** irregular

Lumbar 3d-CT and X-ray were taken preoperatively and postoperatively at 1 day as well as 1, 3, 6, and 12 months postoperatively. Disk height (DH) was measured as the vertical distance between the midpoint of the cranial endplate and the caudal endplate on the 3D-CT midsagittal plane (Fig. 1a). CS is defined as a reduction of more than 2 mm in DH during follow-up, compared to 1 day postoperatively. Patients with DH decrease ≤ 2 mm were classified into the no CS (NCS) group, those with 2 mm < DH decrease ≤ 4 mm were classified into the mild CS (MCS) group, while those with DH decrease > 4 mm were classified into the severe CS (SCS) group [8]. The demographics analysed included sex, age, preoperative diagnosis (with or without degenerative spondylolisthesis), bone mineral density (BMD), and body mass index (BMI). We utilized the minimum T score obtained from the hip using dual-energy X-ray absorptiometry (DEXA) scans, as lumbar spine DEXA information is often inaccurate in patients with lumbar degenerative pathology [11]. The radiological parameters analysed included DH distraction, cage position, endplate sclerosis or injury, disk space morphology, and fusion rate. DH distraction was calculated as the increment of DH at 1 day postoperatively, compared with preoperative DH. The cage position was measured as the percentage of the distance between the anterior metal marker and the leading edge of the caudal endplate; to the length of caudal endplate using X-ray taken 1 day postoperatively (Fig. 2). Disk space morphology was classified on MRI as flat, concave, or irregular according to the criterion described by Pappou et al. [12] (Fig. 3). The clinical outcomes analysed included visual analogue scale (VAS) pain scores of the lower back and leg, and the Oswestry Disability Index (ODI), which were recorded preoperatively and 1, 3 and 12 months postoperatively. Fusion was evaluated using 3D-CT taken 12 months postoperatively according to the criteria described by Bridwell et al. [13]. The clinical outcomes and fusion rates were compared between patients in the three groups. The demographic and radiological parameters were also compared between patients with and without CS.

### Statistical analysis

SPSS 22.0 (IBM Corp., Armonk, New York, USA) software was used for analysis. Chi-squared analysis was performed for categorical variables, and one-way analysis of variance was performed for continuous variables. Significance was set at *P* < 0.05. Univariate binary logistic regression (UBLR) was used to adjust for confounding variables, variables with *P* < 0.15 were allowed to enter the multivariate binary logistic regression analysis (MBLR), and *P* < 0.05 was considered statistically significant for MBLR.

## Results

A total of 242 patients were enrolled in the study. CS was identified in 79 (32.6%) patients, including 48 (19.8%) in the MCS group and 31 (12.8%) in the SCS group. The remaining 163 (67.4%) patients were assigned to the NCS group. There were no significant differences in demographic parameters between the three groups (Table 1).

**Table 1:** Comparative analysis of demographics between three groups

	NCS group	MCS group	SCS group	*P*
Patients (n)	163	48	31	-
Sex (Male: female)	65:98	19:29	14:17	0.851
BMI (Kg/m^2^)	24.7 ± 3.4	25.4 ± 3.1	24.5 ± 3.1	0.342
Age (years)	64.5 ± 9.1	66.3 ± 10.7	69.1 ± 9.9	0.099
Diagnosed with DS (Yes: No)	55:108	22:26	11:20	0.308

Data presented as mean ± standard deviation. *DS* Degenerative Spondylolisthesis

### Characteristics of subsidence

**Fig. 4:**
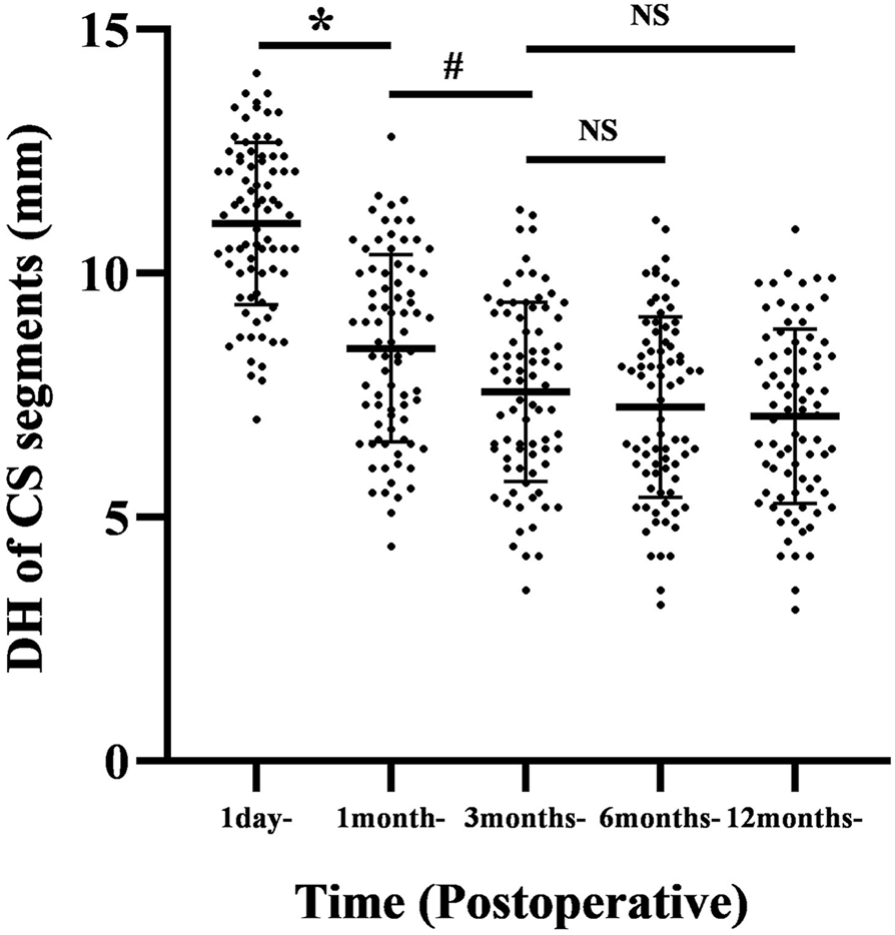
Comparative analysis of the disk height (DH) of the cage subsidence segments at each follow-up. ^*^, *P* < 0.00, 1 month post- versus 1 day post-; ^**#**^, *P* = 0.003, 1 month post- versus 3 months post-; NS, *P* > 0.05, compared to 3 months post-. Post, postoperative

**Fig. 5:**
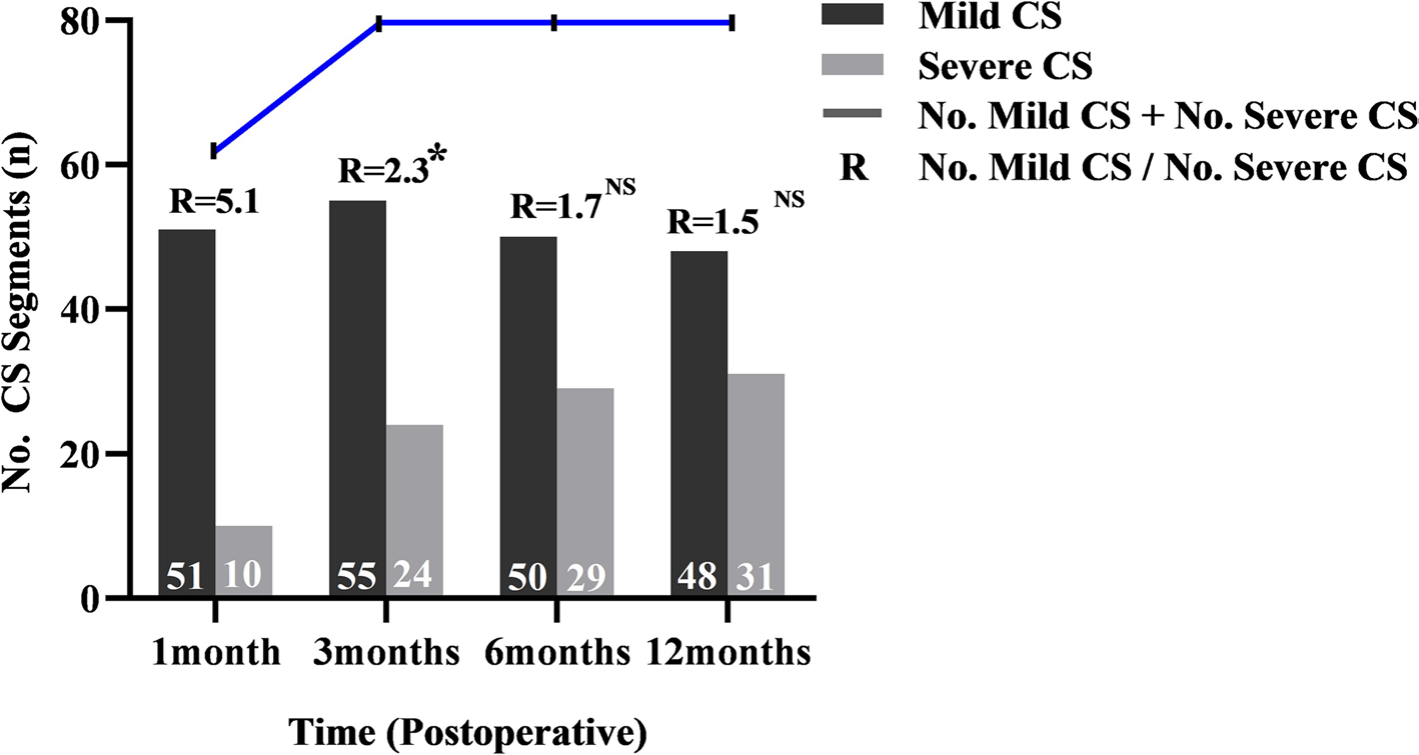
Comparative analysis of the number of mild cage subsidence (MCS) and severe cage subsidence (SCS) segments and their ratio at each follow-up. *, *P* < 0.05, 1 month post- versus 3 months post-; NS, *P* > 0.05, compared to 3 months post-. Post, postoperative. R, the ratio of MCS to SCS segments

The significant DH decrease of the subsidence segments occurred within 3 months postoperatively, from 11.0 ± 1.7 mm 1 day postoperatively to 8.5 ± 2.0 mm 1 month postoperatively (*P* < 0.001) and continued to 7.6 ± 1.9 mm at 3 months postoperatively (*P* = 0.003). Compared with 3 months postoperatively, the DH only slightly decreased to 7.3 ± 1.9 mm (*P* = 0.291) and 7.1 ± 1.8 mm (*P* = 0.084) at 6 and 12 months postoperatively (Fig. 4). At 1 month postoperatively, a total of 61 patients were identified with CS including 51 with MCS and 10 with SCS. This number increased to 79 at 3 months postoperatively, including 55 with MCS and 24 with SCS, indicating a significant decrease in the ratio of MCS to SCS (*P* = 0.042). After 3 months postoperatively, no additional CS occurred. Only 5 and 2 segments developed from MCS to SCS at 6 and 12 months postoperatively, without significant changes in the ratio of MCS to SCS segments (*P* = 0.250, 0.156) compared to 3 months postoperatively. (Fig. 5). A total of 44 CS cases occurred in the caudal endplate, 30 occurred in both caudal and cranial endplates, and the remaining 5 only occurred in the cranial endplate. Typical radiological data is shown in Fig. 1.

### Clinical and radiological results

**Fig. 6:**
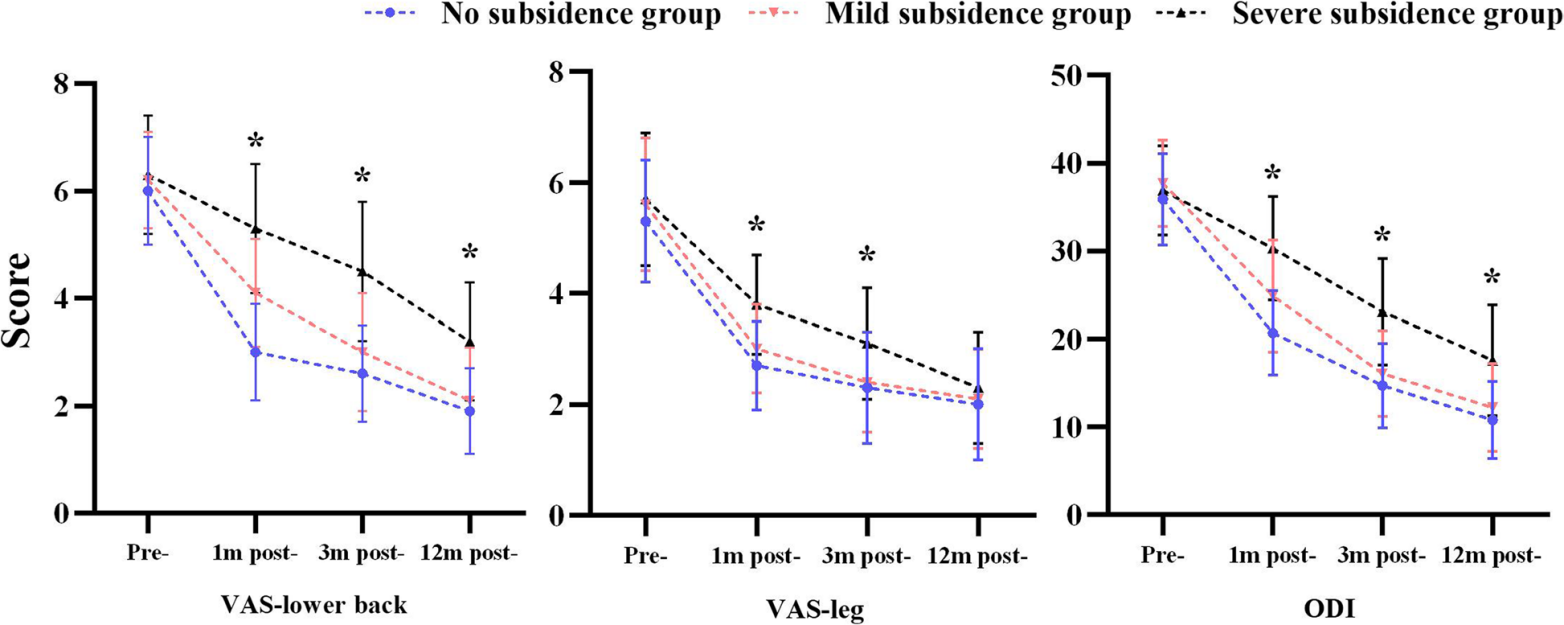
Comparative analysis of clinical outcomes at each follow-up between three groups. *, *P* < 0.05, compared between three groups. Pre-, preoperative; post-, postoperative

The clinical outcomes of three groups are presented in Table 2 and Fig. 6. No significant differences existed in the clinical score between the three groups preoperatively (*P* = 0.069, 0.085, 0.094). At 1 month postoperatively, ODI and VAS scores of lower back in MCS group were significantly higher than those in NCS group (*P* < 0.001), but significantly lower than those in SCS group (*P* < 0.001). There were no significant differences in VAS scores of legs between NCS and MCS groups 1 month postoperatively (*P* = 0.057), and both of them were significantly lower than those in SCS group (*P* < 0.001). At 3 months postoperatively, there were no significant difference in any clinical result between NCS and MCS groups (*P* = 0.064, 0.836, 0.180), and both were significantly lower than those in SCS group (*P* < 0.001). At 12 months postoperatively, ODI and VAS score of lower back in NCS and MCS groups were significantly lower than those of SCS groups (*P* < 0.001), while VAS score of leg was comparable between three groups (P_(NCS-SCS)_ = 0.775, P_(MCS-SCS)_ = 0.724).

**Table 2:** The clinical outcomes between three groups

	NCS group	MCS group	SCS group
VAS-lower back			
Pre-	6.0 ± 1.0	6.2 ± 0.9	6.3 ± 1.1
1 m post-	3.0 ± 0.9^*^	4.1 ± 1.0^*^	5.3 ± 1.2^*^^^^
3 m post-	2.6 ± 0.9^#^	3.0 ± 1.1^#^	4.5 ± 1.3^#**^**^
12 m post-	1.9 ± 0.8^&^	2.1 ± 1.0^&^	3.2 ± 1.1^&**^**^
VAS-leg			
Pre-	5.3 ± 1.1	5.6 ± 1.2	5.7 ± 1.2
1 m post-	2.7 ± 0.8^*^	3.0 ± 0.8^*^	3.8 ± 0.9^*^^^^
3 m post-	2.3 ± 1.0^#^	2.4 ± 0.9^#^	3.1 ± 1.0^#**^**^
12 m post-	2.0 ± 1.0^&^	2.1 ± 0.9^&^	2.3 ± 1.0^&^
ODI			
Pre-	35.9 ± 5.2	37.7 ± 4.9	36.9 ± 5.1
1 m post-	20.7 ± 4.8^*^	24.9 ± 6.4^*^	30.3 ± 5.9^*^^^^
3 m post-	14.7 ± 4.8^#^	16.1 ± 4.9^#^	23.1 ± 6.1^#**^**^
12 m post-	10.8 ± 4.4^&^	12.2 ± 5.0^&^	17.6 ± 6.3^&**^**^

Data presented as mean ± standard deviation. Pre-, preoperative; post-, postoperative

^*^, *P* < 0.01, compared to pre-; ^**#**^, *P* < 0.01, compared to 1 m post-; ^**&**^, *P* < 0.05, compared to 3 m post-., *P* < 0.01, compared to NCS group; ^**^**^, *P* < 0.01, compared to MCS group

The fusion rates were 92.6% (151/163), 83.3% (40/48) and 64.5% (20/31) in NCS, MCS and SCS groups, respectively. No significant differences in fusion rates between NCS and MCS groups (*P* = 0.053) or MCS and SCS groups were observed (*P* = 0.056). However, fusion rate in the SCS group was significantly lower than that in NCS group (*P* < 0.001).

### Risk factors

**Fig. 7:**
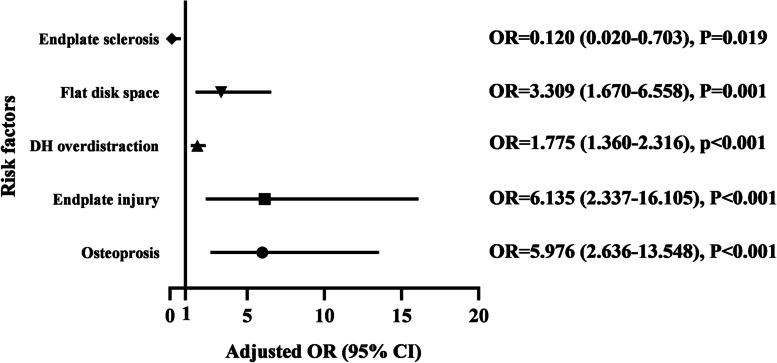
Results of multivariate binary logistic analysis. OR, odd ratio; CI, confidence interval

In UBLR analysis, BMD (*P* < 0.001), distraction of DH (*P* = 0.019), disc space morphology (*P* = 0.044), endplate sclerosis (*P* = 0.075), and endplate injury (*P* = 0.001) were significantly associated with CS, while age, sex, BMI, diagnosis, and cage position were not (Table 3). In further MBLR analysis (Fig. 7), osteoporosis was a significant risk factor for CS (*P* < 0.001) which increased the risk to 5.976 (2.636–13.548) times. Endplate injury occurred in 22.8% (18/79) of CS group, significantly higher than 6.7% (11/163) in NCS group (*P* = 0.001), it was significantly related to CS (*P* < 0.001) and increased the risk to 6.135 (2.337–16.105) times. In addition, the flat disk space and the DH over-distraction were also significantly correlated with the increased probability of CS (*P* < 0.01), the adjusted OR were 3.309 (1.670—6.558) and 1.775 (1.360—2.316), respectively. In contrast, endplate sclerosis showed as a significant protective factor for CS (*P* = 0.019), with the adjusted OR of 0.120 (0.020—0.703).

**Table 3:** Outcomes of univariate binary logistic regression analysis for risk factors

Factors	CS group	NCS group	Rough OR (95% CI)	P
Sex (male: female)	34:45	65:98	1.082:1(0.626–1.867)	0.778
Age (years)	67.1 ± 10.4	64.5 ± 9.1	1.437:1(0.833–2.479)	0.192
BMI (Kg/m^2^)	25.0 ± 3.1	24.7 ± 3.4	1.213:1(0.692–2.128)	0.500
Diagnosed with DS (Yes: No)	33:46	55:108	1.409:1(0.811–2.448)	0.224
BMD (≤ -2.5: > -2.5)	24:51	22:142	3.935:1(2.020–7.668)	< 0.001*
DH distraction (mm)	3.3 ± 1.4	2.9 ± 1.3	1.267 (1.039–1.544)	0.019*
Disk space morphology				
(Flat: Concave)	42:29	59:83	2.037:1(1.142–3.635)	0.016*
(Irregular: Concave)	8:29	21:83	1.090:1(0.436–2.729)	0.835
Endplate injury (Yes: No)	18:61	11:152	4.077:1(1.820–9.136)	0.001*
Endplate sclerosis (Yes: No)	2:77	15:148	3.902:1(0.870–17.503)	0.075*
Cage position	22.8 ± 7.4%	22.4 ± 7.0%	2.735(0.060–124.657)	0.606

Data presented as mean ± standard deviation. *DS* Degenerative Spondylolisthesis

^*^: *P* < 0.15, statistical significance for univariate binary logistic regression analysis

## Discussion

The present study was the first of its kind to investigate the impact of CS on fusion rate and clinical results following OLIF combined with anterolateral fixation. The overall incidence of CS and fusion in our study were 32.9% and 87.2%, respectively, which were within the reported ranges of 10%-40% and 87.2%-97.9% following OLIF combined with bilateral pedicle fixation or stand-alone [3–5].

CS is a progressive development that manifests as cages sinking into vertebrae through adjacent endplates prior to complete fusion [9]. Currently, large variations were reported in the development of CS after LIF technique [9, 14]. Chen et al. [14] presented CS as a late event which was identified at 3 months postoperatively and continuously progressed until 2 years after lateral LIF (LLIF) technique. In contrast, Marchi et al. [9] argued that CS occurred mainly within 6 weeks and without significant progress after 3 months following LLIF. A similar trend was found in this present study, our results suggested that CS following OLIF should be classified as an early complication that occurred primarily at 1 month postoperatively and did not progress significantly after 3 months postoperatively. Therefore, the early postoperative stage should be considered a vital period to address CS after OLIF surgery.

CS has great significance for LIF technique, as it means some surgical goals, such as intervertebral distraction, indirect decompression or alignment correction, may not be met [8, 9]. Various studies have compared CS to surgical results following LIF technique, and a clear relationship was not found [6–9]. Our results indicated that CS were related to surgical results following OLIF. Higher magnitudes of CS were associated with worse surgical improvements. Marchi et al. [9] proposed that low grade CS (DH reduction less than 25%) was the result of endplate remodelling due to the natural curvature of endplate, and does not interfere with subsequent fusion. Similarly, we noted that mild CS yielded a comparable fusion rate compared to no CS group. But it caused a transient poor clinical improvement. We speculated that this poor clinical improvement may be the result of transient local bony changes such as endplate inflammation, and may abate over time after CS stabilizes and correct to similarly improved clinical outcomes. In contrast, we found that severe CS caused not only poor clinical achievements, but also significantly reduced the fusion rate. On the one hand, we inferred that severe CS may aggravate and prolong this bone change, thus causing aggravated and constant poor clinical improvements. On the other hand, with respect to intervertebral fusion, it requires a stable biomechanical environment to promote trabecular connections. We considered that the remarkable reduction in the height of the intervertebral space due to severe CS may result in the re-relaxation of the ligamentous structure at the index level and thus fail to provide a stable biomechanical environment necessary for the fusion process, eventually leading to reduced fusion rate [15]. Based on our aforementioned results, as CS following OLIF was associated with poor surgical improvements, it was helpful to identify the related risk factors so that CS can be prevented.

Risk factors related to CS are multifactorial. Generally speaking, endplate stiffness and the interfacial load between the implant and the endplate are the basic factors affecting the occurrence of CS [16, 17]. Oxland et al. [18] proposed that the endplate stiffness decreased by approximately 33% after injury, thus inducing CS. In our study, we found that endplate injury significantly increased the occurrence of CS, therefore, we suggest that attention should be given to avoiding endplate injury intraoperatively, which may be beneficial in reducing CS. Gentle manipulation, BMD examination and a cage with appropriate height should be recommended to avoid endplate damage. Endplate stiffness also varies with the anatomic region [19]. Hou et al. [19] demonstrated that the failure loading required for CS was maximum when the cage was placed posterolaterally on the endplate with the strongest stiffness. Kim et al. [20] also reported that anterior cage position was a risk factor for CS following transforaminal LIF (TLIF) surgery. However, we failed to find a clear relationship between CS and cage position. We speculate that the position of the cage placed through the oblique channel was overall anterior and the range of anteroposterior position was narrow [21], so it is may not enough to reflect the stiffness discrepancy at different endplate regions. In addition, an early study concluded that the cranial endplate is 40% stiffer than the caudal endplate [22], we confirmed this conclusion in the present study as we found that CS occurred more frequently in the caudal endplate.

BMD was also considered to be a vital factor affecting endplate stiffness. Hou et al. [19] found that decreased BMD resulted in lower endplate stiffness and lower failure loading for CS. Tempel et al. [11] presented that the sensitivity and specificity of a DEXA T score of -1.0 or less for predicting CS following LLIF were 78.3% and 63.2%, respectively. Park et al. [23] calculated that osteoporosis increased the CS risk following TLIF by 4.8-fold. We calculated that the osteoporosis increased the risk by 6.0-fold following OLIF, which was slightly higher than the risk coefficient of CS following TLIF. This finding indicated that stringent constraints may be required for bone condition, to prevent CS following OLIF.

Increased compressive forces is another basic mechanism which result in endplate fracture and CS [24]. DH over-distraction is widely accepted as risk factor for CS in spinal interbody fusion surgery [25]. In our study, we found that the increase in DH distraction was significantly correlated with CS following OLIF. Therefore, selecting appropriate cage height and avoiding excessive DH distraction may be beneficial for CS prevention. At present, the correct methods to select the appropriate cage height are still controversial. Some studies have suggested that the height should be determined according to the DH measured preoperatively [26], while others recommend should be determined by the compressive and distractive force generated by cage implantation [24].

The impacts of disc space morphology on CS have been preliminarily mentioned [23, 27]. Park et al. [23] presented that pear-shaped disc space was more likely to induce CS, interpreted as the smaller contact between the endplate and cage causes a stress concentration, thus inducing CS. Similarly, we found the CS risk significantly increased in cases with flat disc space compared to concave space. Therefore, customizing a specific shape cage according to the disc space shape to increase the match between cage and endplate may be helpful to reduce CS.

### Limitations

We acknowledge that the retrospective study design with short-term follow up is a limitation. Secondly, subsidence is a multidimensional parameter and that its evaluation in a single plane, as in this study, may not be sufficient to evaluate its characteristics. Thirdly, there are biomechanical differences between anterolateral fixation and pedicle screw fixation, thus our results are only applicable to OLIF combined with anterolateral fixation. Also, as a reflection of the effectiveness of anterolateral fixation, the information of screw displacement was missing. Finally, other factors that may affect CS, such as cage length and lumbar lordosis, were not evaluated in this study and should be further investigated.

## Conclusion

This investigation reviewed the impacts of CS on OLIF surgery, and further investigated its characteristics and related risk factors. We concluded that CS following OLIF surgery should be considered as an early postoperative complication. Higher magnitudes of CS were associated with worse clinical improvements and lower intervertebral fusion. Osteoporosis and endplate injury were significant risk factors for CS. In addition, a flat disk space and DH over-distraction were also correlated with CS. Attention should be closely paid to eliminate related risk factors to avoid CS when performed OLIF surgery.

## Data Availability

Available via the corresponding author’s email when the manuscript is received:

## Abbreviations

LIF: Lumbar interbody fusion
OLIF: Oblique lumbar interbody fusion
LLIF: Lateral lumbar interbody fusion
TLIF: Transforaminal interbody fusion
CS: Cage subsidence
NCS: No cage subsidence
MCS: Mild cage subsidence
SCS: Severe cage subsidence
DH: Disk height
BMD: Bone mineral density
BMI: Body mass index
DEXA: Dual-energy X-ray absorptiometry
VAS: Visual analogue scale
ODI: Oswestry Disability Index
UBLR: Univariate binary logistic regression
MBLR: Multivariate binary logistic regression
